# 3D Printed Models in Cardiology Training

**DOI:** 10.1016/j.jacadv.2024.100893

**Published:** 2024-02-28

**Authors:** Devin Chetan, Israel Valverde, Shi-Joon Yoo

**Affiliations:** a Division of Cardiology, Labatt Family Heart Centre, The Hospital for Sick Children, Toronto, Ontario, Canada; b Department of Pediatrics, University of Toronto, Toronto, Ontario, Canada; c Division of Cardiac Imaging, Department of Diagnostic Imaging, The Hospital for Sick Children, Toronto, Ontario, Canada; d Department of Medical Imaging, University of Toronto, Toronto, Ontario, Canada

**Keywords:** cardiac 3D printing, cardiology education, cardiology training, virtual reality

3 Dimensional (3D) printing has emerged as an innovative technique with significant importance in congenital heart surgery. It offers opportunities for improving surgical planning, precision, and patient outcomes. Surgeons can then use these models to visualize complex anatomical structures and relationships and develop personalized surgical strategies. This improves preoperative planning and allows for the customization of surgical procedures to meet patient needs with the added benefit of hands-on surgical training, both for trainees learning and practicing complex operations and for more experienced surgeons refining their approach.^1^

Although educational exposure to advanced imaging, specifically cardiac 3D models, has been gaining traction in cardiology, we recognize the considerable advantages of introducing cardiac 3D models at an earlier stage of the training process. Early exposure would afford trainees a deeper appreciation for the intricacies of complex anatomical structures and their relationship to one another, an improved understanding of cardiac imaging including echocardiography, computed tomography, and magnetic resonance imaging, and a better ability to formulate appropriate treatment plans for patients. Although cardiac 3D models have the potential to significantly advance understanding of pediatric cardiology, we believe integration into the training curriculum is currently inadequate and warrants reconsideration.

Virtual reality (VR) offers different benefits including reduced cost, ability to visualize from different angles or orientations, ability to communicate with others in the same simulated environment, and options for device fitting or other procedural planning with the ability to simulate grafts, baffles, stents, or other implants. While the focus of this article is on 3D models, we include references to VR applications where relevant. We sought to review the literature outlining the utility and applications of cardiac 3D-printed models and provide our perspective on current and future uses for cardiology training.

## Morphology teaching and comparison to pathologic specimens

While pathologic specimens, which have traditionally been used for teaching, are valuable tools, they carry their own set of limitations. Namely, there are ethical, legal, and cultural concerns with respect to cadaveric specimens,^2^ and programs will have access to different quantities and quality of specimens based on how their collections were accrued. With improvements in outcomes, pathologic specimens will become increasingly scarce. Furthermore, they are prepared in a certain way that can show some, but likely not all, features that may be of importance. Specimens must also be stored appropriately, which can limit their availability for trainee use. Finally, specimens are subject to wear and tear from repeated handling over time.

Many of these limitations can be overcome with cardiac 3D-printed models. While not inexpensive, cardiac 3D models democratize access to this important educational tool. Namely, regardless of program size or resources, trainees can have access to a full gamut of specimens with a complete range of cardiac pathology. This includes simple pathology that is important to learn but may be hard to find as a pathologic specimen and advanced pathology that may be extremely rare but carries important teaching concepts for the trainee. Relevant features of the anatomy or morphology can be demonstrated from an almost infinite number of angles or planes to highlight important features. Models are generally easy to maintain, and once printed, they can be used repeatedly with minimal wear and tear. Models also do not have specific storage requirements, meaning they can be made more available for regular trainee use. Despite their numerous advantages, there are still important limitations in demonstrating valvular or myocardial pathology using 3D-printed models. This limitation may be overcome with VR.^3^

## Utility in cardiology education

Beyond the surgical domain, cardiac 3D models have been used in nursing, medical student, and resident education.^4^, ^5^, ^6^ While some studies have shown promise in terms of improving spatial orientation and structure identification, others have failed to show a significant long-term impact.^7^, ^8^, ^9^ We feel this may be due to studies that are underpowered to adequately assess the outcome of interest or possibly, that students require more repeated longitudinal exposure to truly benefit from the enhanced spatial understanding. While there is a paucity of literature demonstrating the impact of using 3D printed models on education for cardiology fellows, there has been some promising evidence suggesting it can be helpful across the spectrum from medical students to consultants in pediatric cardiology.

### Anatomy and imaging

A review from Anwar et al^10^ outlined the primary utility of cardiac 3D models for education by reducing the learning curve for trainees by improving their understanding of complex 3D anatomy, providing high-fidelity simulation experiences, and increasing exposure to rare pathology. Similarly, another review highlighted the importance of 3D printing for education by improving engagement to achieve a faster understanding of anatomical defects and by exposing students to a broad spectrum of malformations with significant variability that can exist even within the same condition.^2^

Valverde et al examined 127 participants and assessed their understanding of criss-cross heart using conventional imaging methods (echocardiography and cardiac magnetic resonance imaging) compared to 3D-printed models. There was a significant improvement in anatomical knowledge overall, for each individual case presented and in each group (medical students, pediatric cardiology fellows, and pediatric cardiology consultants). Participants rated their experience with 3D models, and overall, participants felt 3D models improved their understanding of congenital heart disease, increased their interest in anatomy, improved their understanding of imaging, should be included in medical workshops, could replace cadaveric specimens for teaching, and were useful for communication with patients.^11^

At our institution, our 3D printing program has worked with the intensive care unit to develop models of basic echocardiography views along with associated sessions for critical care trainees learning point-of-care ultrasound. Although not formally evaluated with a study, the sessions have received excellent ratings from the trainees, who felt the models enhanced their spatial understanding. Similarly, a study from Costello et al^12^ showed improvement in knowledge acquisition, knowledge reporting, and structural conceptualization of ventricular septal defects amongst 23 pediatric residents learning with cardiac 3D models.

### Interventional procedures

Although data supporting training in interventional cardiology is scarce,^13^ cardiac 3D models have been used in simulation. Valverde et al demonstrated feasibility with a 3D-printed model used for planning endovascular stenting in transverse arch hypoplasia. Both participants in the study found the model helpful in planning the intervention and improving communication, and they felt it had the potential to reduce the risk of catheterization complications.^14^ Brunner et al^15^ studied 19 participants with various levels of cardiac catheterization experience, ranging from none to fully trained pediatric cardiologists with extensive experience in the catheterization laboratory. Fluoroscopy times decreased with practice, and most participants agreed that the 3D models were useful for learning the steps of a catheter intervention, were well suited for learning how to use catheterization devices, and agreed that more opportunities to practice on 3D models would help in achieving improved patient safety.^15^ While evidence for use is currently limited, the Society for Cardiovascular Angiography and Intervention’s Simulation Committee feels simulation is likely to take on a bigger role in training and maintaining certification.^13^

### Communication

Cardiac 3D models have the potential to improve communication with patients and their families. This visual representation can help grasp important complexities and better understand treatment options, potential risks, and alternatives. Preliminary experience from a single center showed that caregivers found the models very helpful in improving anatomical understanding.^10^ A study from Biglino et al showed that although there was no statistically significant improvement in short-term parent understanding, both parents and cardiologists found the models helpful in improving engagement and communication. As there is a transition to a greater emphasis on competency-based education in cardiology, there is a need for learners to present information to families that is specific to their condition and appropriate for their understanding based on their needs and background. Cardiac 3D models are one option in their toolkit that can help with sharing information, engaging patients and families, strengthening relationships with families, and promoting greater involvement in healthcare through shared decision-making.^16^

## Limitations

One significant challenge lies in the development and accessibility of this technology. Creating high-quality, detailed cardiac models demands not only specialized knowledge but also substantial resources like equipment and expertise. Access is improving through shared libraries (eg, NIH 3D), VR (where applicable), and/or commercially available education sets (eg, 3D PrintHeart). Another concern is the issue of reimbursement, as many institutions are navigating the complex landscape of reimbursement policies for incorporating 3D-printed models into their educational curriculum or clinical practice. Collaborative efforts amongst centers skilled in 3D printing and the pediatric cardiology/radiology community will hopefully help make these tools more widely available through advocacy efforts to ensure fair reimbursement for time and resources spent developing these programs.

## Conclusions

Based on the literature presented, it is evident that there should be an increased focus on integration of 3D-printed models in congenital heart disease education. The utility of these models transcends mere anatomical comprehension, encompassing vital aspects such as imaging training, simulation exercises, and effective communication.^2^ Institutions should be proactive in fostering collaboration while remaining vigilant about potential challenges. Establishing clear guidelines for responsible sharing that promote educational access while maintaining transparency and accountability is a worthwhile objective.

## Funding support and author disclosures

The authors have reported that they have no relationships relevant to the contents of this paper to disclose.
